# The South American monsoon approaches a critical transition in response to deforestation

**DOI:** 10.1126/sciadv.add9973

**Published:** 2023-10-04

**Authors:** Nils Bochow, Niklas Boers

**Affiliations:** ^1^ Department of Mathematics and Statistics, Faculty of Science and Technology, UiT The Arctic University of Norway, Tromsø, Norway.; ^2^ Physics of Ice, Climate and Earth, Niels Bohr Institute, University of Copenhagen, Copenhagen, Denmark.; ^3^ Potsdam Institute for Climate Impact Research, Potsdam, Germany.; ^4^ Earth System Modelling, School of Engineering and Design, Technical University of Munich, Munich, Germany.; ^5^ Department of Mathematics and Global Systems Institute, University of Exeter, Exeter, UK.

## Abstract

The Amazon rainforest is threatened by land-use change and increasing drought and fire frequency. Studies suggest an abrupt dieback of large parts of the rainforest after partial forest loss, but the critical threshold, underlying mechanisms, and possible impacts of forest degradation on the monsoon circulation remain uncertain. Here, we use a nonlinear dynamical model of the moisture transport and recycling across the Amazon to identify several precursor signals for a critical transition in the coupled atmosphere-vegetation dynamics. Guided by our simulations, we reveal both statistical and physical precursor signals of an approaching critical transition in reanalysis and observational data. In accordance with our model results, we attribute these characteristic precursor signals to the nearing of a critical transition of the coupled Amazon atmosphere-vegetation system induced by forest loss due to deforestation, droughts, and fires. The transition would lead to substantially drier conditions, under which the rainforest could likely not be maintained.

## Teaser

There is evidence that the South American Monsoon is approaching a critical transition due to deforestation and land-use change.

## INTRODUCTION

In combination with the impacts of anthropogenic climate change, regional land-use changes during the past decades have posed an unprecedented threat to the Amazon rainforest. Several studies suggest the existence of critical thresholds for an Amazon dieback and transition to savanna state, at global warming of 3° to 4°C on the one hand and deforestation of around 40% of the original rainforest area (*1*–*8*) on the other hand. Global warming has recently crossed 1°C above preindustrial levels, and more than 20% of the Brazilian Amazon rainforest area has already been cleared (*9*). Crossing the critical thresholds might induce an abrupt dieback of large parts of the rainforest, with severe ecological and climate impacts from regional to global scales.

The Amazon naturally provides many ecosystem and climate services (*10*) and is an essential component of the Earth’s hydroclimate (*11*, *12*). It constitutes the Earth’s largest terrestrial carbon sink (*13*–*15*) and is essential for local and regional climate stability (*16*), with predicted decreases in precipitation and increases in air temperatures in South America in response to an Amazon dieback (*5*, *17*–*22*). Besides the critical threshold in the vegetation system (*3*), a potential tipping point in the coupled atmosphere-vegetation dynamics of the South American monsoon system and the Amazon rainforest has been proposed, with serious implications for the monsoon circulation system when deforestation rates exceed 30 to 50% (*5*, *23*).

Considering the potential consequences of an Amazon dieback, there is still a substantial lack of studies investigating potential precursor signals for critical transitions of the coupled vegetation-atmosphere system of tropical South America. There is growing empirical evidence of climatological and hydrological changes in Amazonia, such as rising air temperatures, extended dry seasons, more frequent hydrological extreme events, particularly droughts, and increasing soil moisture stress. While previous droughts in the Amazon basin have partially been associated with changes in the regional circulation due to changes in the Hadley cell (*24*–*27*), the precise connection of all these changes with deforestation remains a largely open problem (*22*, *28*–*31*).

On the basis of a space-for-time replacement, it has been shown that temporal autocorrelation of vegetation data [normalized difference vegetation index (NDVI)] scales negatively with mean annual precipitation in tropical forests, which suggests that rainforest vegetation is less resilient in more arid regions (*32*, *33*). Furthermore, a loss of resilience of large parts of the rainforest over the past decades has been observed using remotely sensed vegetation optical depth data (*34*). Changes in the temporal autocorrelation can, under certain conditions, be interpreted as indicative of a loss of stability; however, such an analysis has not yet been performed for the coupled Amazon vegetation-atmosphere system.

Here, we show that observed changes in the hydrological cycle, as well as characteristic changes in well-established statistical indicators, might be first warning signals of a forthcoming regime shift.

For this purpose, we investigate several indicators that are associated with critical slowing down (CSD) when a system approaches a bifurcation-induced transition (*35*–*38*). When approaching the critical threshold, the restoring forces of the system diminish because the basin of attraction widens and thus the recovery rate after perturbations decreases and eventually approaches zero. Fluctuations increase and the state of the system resembles more and more its past states, and the resulting CSD can be traced, e.g., in terms of increasing variance and lag-one autocorrelation, which constitute the classical statistical precursor signals of critical transitions (*36*).

To understand where not only such statistical but also physical process-related precursor signals should be searched for, we first propose a nonlinear model of the moisture transport across South America, based on the fundamental moisture balance equations and coupled to the Amazon vegetation system. Guided by the results of our model simulations, we then conduct a thorough search for statistical and physical precursor signals in the ERA5 reanalysis data. We emphasize that the occurrence of characteristic changes in CSD indicators, such as rising lag-one autocorrelation or variance, on their own by no means implies the possibility or presence of a critical transition or of multistability in the system. After all, there may be many reasons why variance or lag-one autocorrelation might increase, also in systems that are monostable and thus cannot exhibit transitions between alternative states. The theory of CSD should only be applied for anticipating potential critical transitions if there are—a priori and independently from the CSD analysis—good reasons to assume that a given system under investigation has the potential for multistability. On the other hand, however, it should also be noted that, even if a system should not be assumed to exhibit multistability, CSD indicators may still be useful to quantify the resilience of the system (*39*).

In the case considered here, we will show that our model of the moisture transport and recycling over the Amazon suggests, in line with previous studies, that the coupled Amazon atmosphere-vegetation system exhibits the potential for bistability. In turn, the model results imply the potential for critical transitions between alternative states, due to positive feedbacks associated with the latent heating over the Amazon basin and the low-level circulation from the Atlantic Ocean to tropical South America (*5*). In particular, this is suggested independently from any CSD analysis or search for physical precursor signals. Under the a priori assumption of the possibility of alternative stable states as motivated by the modeling results, we then search for statistical CSD-based and physical precursor signals for transitions between the states.

Large parts of the Amazon rainforest experience strong seasonality in the rainfall rates. South of the Equator, the wet season retreat is marked by an abrupt precipitation decline during austral fall (March to May), while the wet season onset is characterized by a rapid increase in rainfall during austral spring (September to November). The wet season initiation has been associated with increased evapotranspiration and large-scale dynamic lifting due to cold-front incursions (*40*–*42*) and monsoon dynamics (*43*) a few months before the Intertropical Convergence Zone (ITCZ) migrates southward, and large amounts of moisture are transported from the tropical Atlantic Ocean to tropical South America. A delay of the wet season onset and increasing fire counts have been linked to changes in atmospheric circulation patterns toward conditions more characteristic for austral winter during the transition season (July to October) in the past decades (*44*). These changes are characterized by a strengthening South American low level jet, increased atmospheric subsidence over southern tropical South America, and fewer southerly cold-air incursions and anomalous convective activity over southern tropical South America (*44*).

**Fig. 1.**
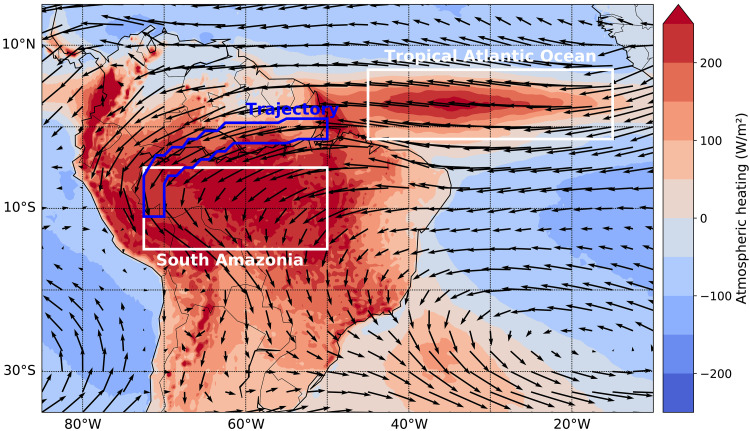
Map of South America with wind fields and vertically integrated heating. Mean wind fields at the 750-hPa level and mean vertically integrated atmospheric heating, i.e., the sum of latent, sensitive, and radiative heating, for the wet season (December to January) from 1979 to 2019. The tropical Atlantic Ocean box and the southern Amazonian box used for our analysis are delineated in white, while the trajectory used for simulations is delineated in blue.

Furthermore, it has been shown that the wet season onset is accelerated by atmospheric processes initiated by increased transpiration in the late dry season (*45*). The beginning of the wet season and the dry season length (DSL) are thus directly linked to the vegetation system. Moreover, convective latent heating over the Amazon basin strengthens the heating gradient between the Atlantic Ocean and the continent (Fig. 1). It has been shown that this enhances the easterly moisture inflow into South America by a factor of 2 to 3 during the wet season (*46*–*48*), establishing a positive feedback in the monsoonal circulation system.

To investigate the effects of deforestation and forest loss due to droughts and fires on the coupled vegetation-atmosphere system and particularly to provide guidance in the search for precursor signals for a critical transition, we extend a recently introduced nonlinear model (*5*) that is based on the fundamental moisture balance equations for the moisture content in the soil and the atmosphere in dependence of precipitation, evapotranspiration, atmospheric advection, and the radiation budget. The model includes a nonlinear contribution representing the acceleration of low-level moisture flow caused by latent heating over the Amazon. Widespread deforestation and drought- or fire-induced forest dieback, via their effects on transpiration rates and radiation, have been shown to potentially trigger a collapse of the positive feedback related to convective latent heating, resulting in abrupt reductions in rainfall amounts after a critical deforestation threshold around 40% is crossed (*5*, *21*, *23*). This effect can already be observed in regions with high forest cover changes of 40 to 50%, with a growing tendency to become water limited due to decreased rainfall, increased potential evapotranspiration, and decreased actual evapotranspiration (*49*).

We extend the model by incorporating a mechanism for the vegetation response to changing precipitation, accounting for increased plant water stress with decreasing precipitation rates. Reduced rainfall rates over longer periods lead to a soil moisture deficit (SMD) and, ultimately, to increased tree mortality with threshold behavior (*50*–*52*). The seasonal cycle of the monsoon system is modeled as a periodically forced bistable system that annually oscillates between an on-state (wet season) and off-state (dry season) with a fold bifurcation–induced transition between on- and off-state (*53*).

Previous studies that investigated moisture recycling across the Amazon basin have not taken into account that changes of the forest functioning alter large-scale wind patterns although these are highly relevant for moisture recycling (*54*–*58*). In the used statistical models, the atmosphere in terms of low-level winds is constant by construction, and, therefore, the aforementioned feedbacks associated with circulation changes are suppressed. The novelty of our approach is to model the winds dynamically to account for these feedbacks between forest loss and alteration of atmospheric circulation patterns. We note that, while changes in the atmospheric circulation in tropical South America in the past decades have been identified, there remain many open questions regarding the links to anthropogenic forest loss (*44*).

Our model is mainly intended to provide guidance in the search for statistical and physical precursor signals in observation-based data, to find out whether the coupled Amazon vegetation-atmosphere system has been approaching a critical threshold. The model is not intended to obtain quantitatively realistic estimates of the consequences of deforestation, for example, in terms of the precise reductions of precipitation. Moreover, our model shall reveal mechanistic reasons for changes in observation-based data rather than to investigate the moisture recycling in Amazonia in detail. By construction, the abruptness of the transition might be overestimated by our model. For example, changes in surface roughness length in response to deforestation, which are ignored in our model, may have attenuating effects regarding the magnitude and the abruptness of the transition.

Previously proposed models (*54*, *56*–*58*) are arithmetic models that compute the overall sum of ingoing and outgoing moisture, in contrast to our proposed model of dynamical differential equations that allow us to study the dynamical behavior of the system. The effects of changing temperature and atmospheric CO_2_ level on the hydrological cycle in Amazonia remain still uncertain, and we do not consider them in our model. A detailed description of the extended model can be found in Materials and Methods.

## RESULTS

To validate our model, we first compare the simulated spatiotemporal evolution of the model variables with corresponding time series from the ERA5 reanalysis (*59*). Across the 100 spatial boxes, we use for our model simulations, spanning the Amazon basin from east to west (Fig. 1), we find a very good agreement of the modeled observables with the corresponding reanalysis data in the wet season. We are also able to reproduce the seasonality, although there are expectable discrepancies between modeled variables and the reanalysis in the dry season (fig. S1).

To investigate the impacts of deforestation on the coupled vegetation-atmosphere system, we integrate our model with a gradually increasing number of deforested boxes. Note that deforestation here refers to either direct forest clearance or forest loss due to droughts or fires. Deforestation starts in the easternmost box and ends in the westernmost box. Deforestation is quantitatively implemented as a 40% decrease in the evapotranspiration and 40% increase in the sensible heat of the respective box, following results from field experiments and regional climate modeling experiments (*60*, *61*). The precipitation recycling rate has been shown to gradually increase from the eastern to the western parts of Amazonia; it is largest in southwestern Amazonia, with more than 50% during the dry season and up to 40% during the wet season (*61*). We therefore use 40% as an annual mean along the trajectory.

**Fig. 2.**
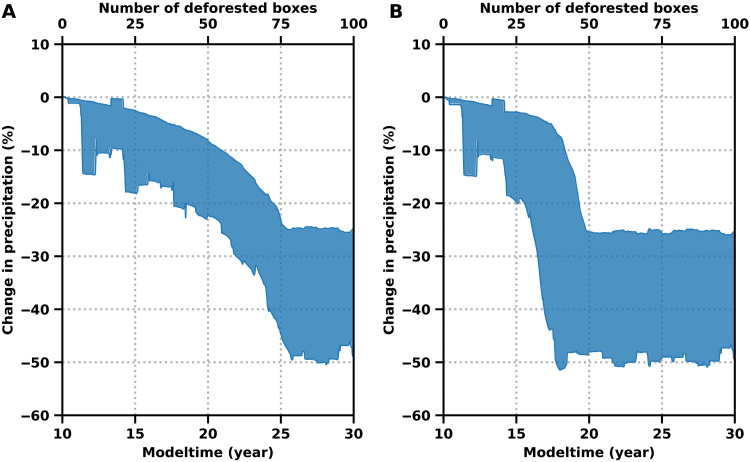
Simulated precipitation rates without and with vegetation feedback after successive deforestation at the end of the trajectory. (**A**) Simulated annual mean precipitation rates, as functions of advancing deforestation at the end of the trajectory (box 90) for all possible choices of the different simulation parameters AF (amplification factor) and ⟨*H*⟩^AO^ (see Materials and Methods) without vegetation feedback. The mean is calculated using a rolling window with size *w* = 1 year for every parameter configuration. Deforestation starts at year 10 in box 0 and subsequently proceeds westward, where it is completed at year 30 in box 100. The ranges of the different simulation parameters are AF = 1.5 to AF = 3, ⟨*H*⟩^AO^(*t*) = (80 ± 40) W/m^2^ to ⟨*H*⟩^AO^(*t*) = (140 ± 40) W/m^2^, with a step width of 0.25 for AF and 20 W/m^2^ for ⟨*H*⟩^AO^(*t*). (**B**) Same as (A) but with vegetation feedback included, leading to early and more abrupt decline of *P*. Although the critical transition is in quantitative accordance with previous studies, we want to emphasize that our model is rather built to give guidance where to search for CSD to anticipate a forthcoming transition.

We allow the system to adapt to the changing number of deforested boxes and reach its equilibrium for each number of deforested boxes, by integrating the model in hourly time steps over time spans of multiple decades. We find a steady but moderate decrease in the rainfall rates at the western boundaries of the Amazon as soon as deforestation in the eastern Amazon sets in. After exceeding a specific threshold in terms of deforested boxes, an abrupt rainfall decline is apparent, which is associated with a bifurcation-induced critical transition in the underlying dynamics, resulting from the collapse of the latent-heat feedback. The system switches into a permanent dry season state and is not able to switch back into the annual wet season. This permanent dry season state is characterized by precipitation rates throughout the year similar to what is presently observed during the dry season. By including the vegetation’s response to increasing water stress, the threshold is crossed considerably earlier, and the subsequent rainfall decline is more abrupt, compared to the original model without vegetation feedback (Fig. 2). This can be explained by the cascading effect introduced by the two-way coupling between the vegetation and the atmosphere (*54*, *56*, *62*). Although the critical forest loss threshold in our model (Fig. 2B) agrees with previous studies, we emphasize that our model is not intended to give quantitatively correct predictions of the critical deforestation level but rather to be used to infer what kind of precursor signals should be searched for in observation-based data to anticipate a critical transition of the couple Amazon atmosphere-vegetation system.

**Fig. 3.**
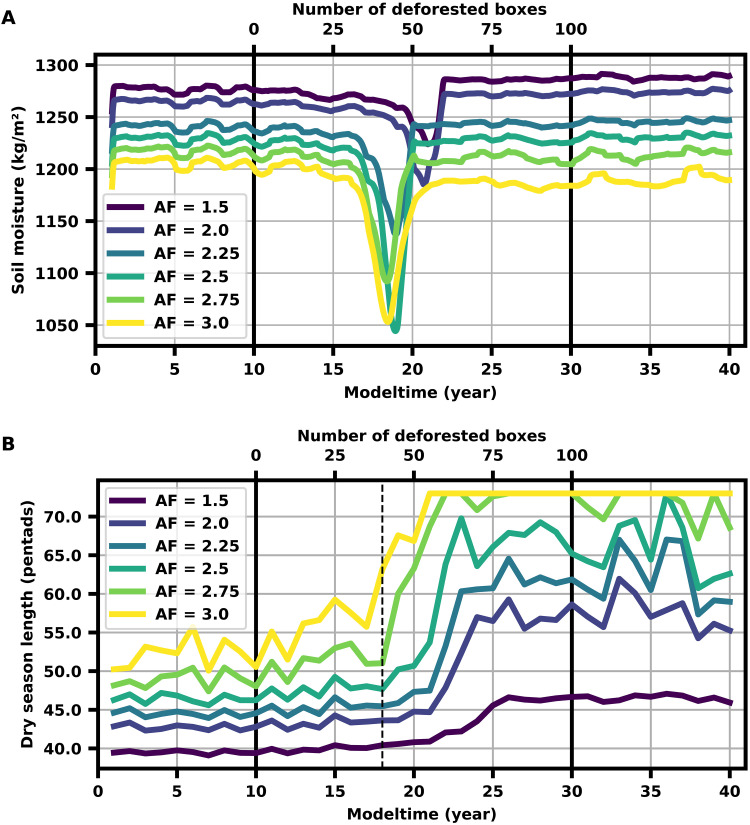
Physical early-warning signals in simulated evolution of soil moisture and DSL in southern Amazonia. (**A**) Time-evolving yearly average of the simulated soil moisture (rolling window with width *w* = 1 year) for different AFs of the low-level moisture flow to South America, integrated SMD threshold = 450,000 kg h/m^2^ and fixed ocean heating ⟨*H*⟩^AO^(*t*) = (120 ± 20) (see Materials and Methods). Deforestation is initiated at year 10 (first solid black line) and completed at year 30 (second solid black line). Note that the soil moisture is the same long before and after the bifurcation-induced transition but declines strongly before the transition when the coupled system loses its prior equilibrium. The shortened wet season and the decreasing rainfall rates result in lower soil moisture levels, while the evapotranspiration rates stay at a high level and further deplete the soil during the dry season. This leads to an all-year-round decrease of the soil moisture and an increasing SMD before the dieback of the rainforest. It therefore serves as an efficient physical precursor for the transition. (**B**) Simulated DSL (*P* < 180/month) with advancing deforestation for same simulation parameters as (A), at the end of the trajectory (box 90). Note the gradual increase of the DSL with an increasing fraction of deforested rainforest before the abrupt transition (dashed black line). For small AF, the switch into the wet season is still possible. However, the wet season length is strongly shortened and wet season precipitation is reduced. The DSL together with the wet season onset (see fig. S3) can, hence, serve as physical precursor signals.

**Fig. 4.**
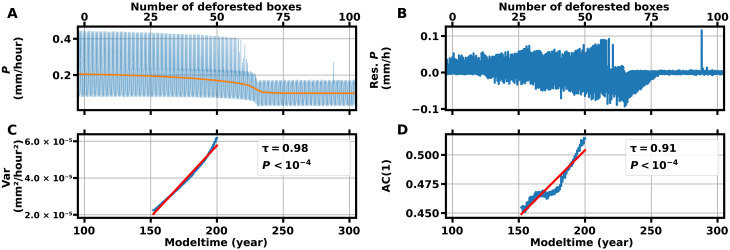
Statistical precursor signals for simulated precipitation rates. (**A**) Five-day averaged simulated precipitation rates, as functions of advancing deforestation at the end of the trajectory (box 90). The nonlinear trend (orange) is obtained using a standard decomposition method (STL) (*95*) with a trend smoother length of 11 years and seasonal smoother length of 13 months. Deforestation starts at year 100 in box 0 and subsequently proceeds westward where it is completed at year 300 in box 100. Before crossing the tipping point, the wet season (*P* ≈ 0.4) and dry season (*P* ≈ 0.15) are apparent. The wet season rainfall rates show a steady decline with advancing deforestation. Flickering and increased variability in the wet season rainfall near the critical threshold can be observed. After exceeding the critical deforestation threshold, only the dry season state remains, with a small seasonal amplitude in the rainfall rates. The chosen simulation parameters are AF = 3, integrated SMD threshold = 20 × 10^6^ kg hour/m^2^, and ocean heating ⟨*H*⟩^AO^(*t*) = (120 ± 20) W/m^2^. (**B**) Residual precipitation time series, obtained by subtracting the seasonal and nonlinear trend components from the original series. (**C**) Time-evolving endpoint variance computed in rolling window of width *w* = 150 years from year 0 (not shown) to year 200 of the detrended time series. Red line denotes the linear trend, and Kendall τ indicates the strength of the increase. (**D**) Same as (C) but for the lag-one autocorrelation [AC(1)].

Before crossing the critical threshold, we observe prominent changes in several hydrological variables and statistical CSD indicators that may serve as precursor signals. Besides a decline in the precipitation *P*, we observe a nonlinear decline in the soil moisture *S* long before the critical transition (Fig. 3A). Furthermore, we find an increase in the DSL that is associated with a later onset of the wet season (Fig. 3B). The wet season initiation is hindered by reduced differential heating between the Atlantic Ocean and the South American continent due to the deforestation-induced decrease in the latent heating over Amazonia. The shortening of the wet season and the concomitant reduction of rainfall rates lead to increased plant water stress, which, in our simplified model, ultimately, triggers the dieback of the remaining rainforest further west. We refer to all the abovementioned pretransition changes in the hydrological cycle and atmospheric circulation as physical precursor signals because they are mechanistically associated with the dynamics leading to the subsequent critical transition, as shown by our modeling results. We choose this term in contrast to the generic, statistical precursor signals associated with CSD preceding critical transitions. We further investigate these well-established indicators, given by the variance and the lag-one autocorrelation of the 5-day averaged modeled precipitation time series, with a deforestation rate of 0.5% per year within a rolling window of 150 years (Fig. 4). We calculate the Kendall rank correlation coefficient τ to measure the strength of the trends and generate surrogate data of the CSD indicators to test the significance of the inferred values of τ. The variance and lag-one autocorrelation show a clear positive trend (τ = 0.98, *P* < 10^−4^, and τ = 0.91, *P* < 10^−4^, respectively; see Fig. 4, C and D, and and fig. S2) and begin to increase long before the transition.

**Fig. 5.**
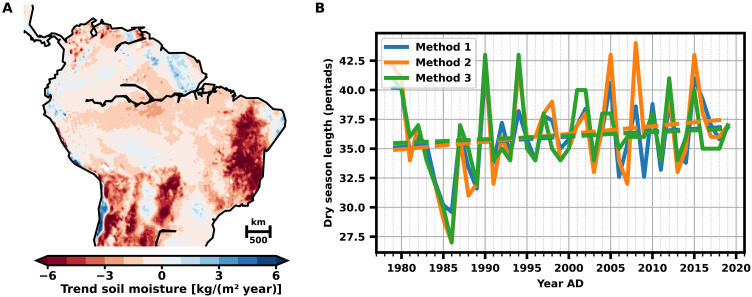
Physical early-warning signals in ERA5 data: evolution of soil moisture and DSL in southern Amazonia. (**A**) Calculated linear trend in yearly averaged soil moisture from 1979 to 2019 in South America, determined from the ERA5 reanalysis for all available soil moisture layers. Most parts of tropical South America show a negative trend (red) in the soil moisture. (**B**) Observed DSL in southern Amazonia (5°S to 15°S, 50°W to 70°W) from 1979 to 2019, determined in three different ways (see Materials and Methods). Dashed lines denote the corresponding linear trends. We find a DSL increase of 4.4 (blue), 6.6 (orange), and 3.0 (green) pentad/century for the three different methods to determine the DSL, respectively. The data are taken from the ERA5 reanalysis (*59*). For a spatial analysis of the DSL, see fig. S4. AD, Anno Domini.

These results give guidance in where to search for precursor signals in the reanalysis and observational data. We analyze all abovementioned, physical and statistical, precursor signals in the evolution of hydrological variables from the ERA5 reanalysis data. We first analyze the soil moisture content and reveal a substantial decline not only in the Amazon basin but also further downstream of the low-level flow in the South American subtropics (Fig. 5A).

We proceed with analyzing the DSL from 1979 to 2019 in southern Amazonia (5°S to 15°S, 50°W to 70°W), using the daily averaged ERA5 reanalysis data using three different methods (see Materials and Methods). We find an extension of the dry season with all three methods, ranging from 1.25 to 2.7 pentads over the past 40 years (Fig. 5B). The main contribution to the lengthening of the dry season is a delayed onset of the wet season, while the wet season demise is relatively constant and only shows a slight tendency toward earlier days of the year (fig. S3). A spatial analysis reveals a significant extension of the dry season primarily in southeastern Amazonia (fig. S4), where deforestation and drought- and fire-induced forest loss are most pronounced (*63*). The increasing DSL and delayed wet season onset in southern Amazonia revealed here corroborate the results of several earlier studies (*25*, *29*, *44*, *64*–*68*). However, it should be noted the different studies do not necessarily agree on the exact spatial distribution of the observed changes.

**Fig. 6.**
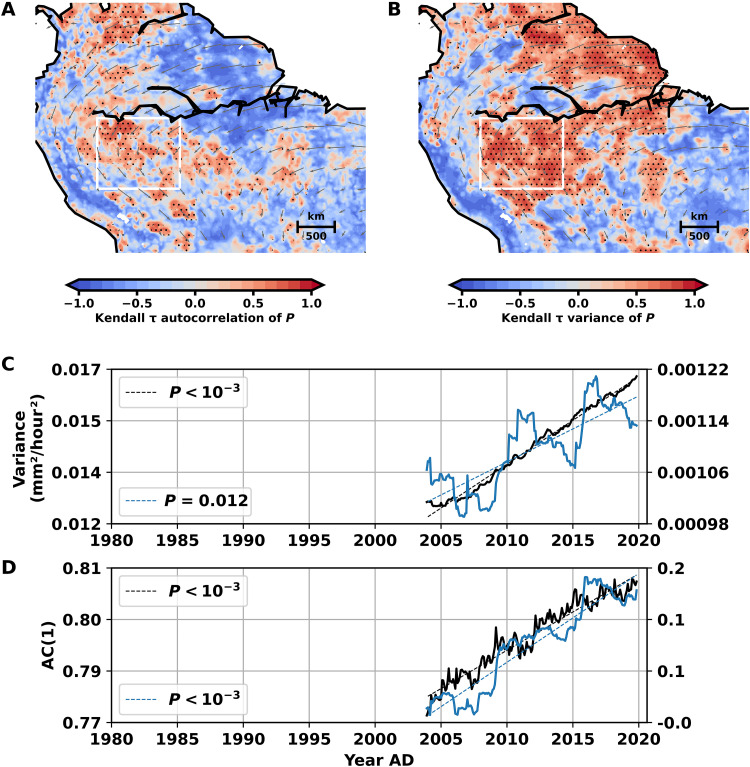
Statistical early-warning signals in ERA5 data. Spatial patterns of Kendall τ for the (**A**) lag-one autocorrelation, and (**B**) variance of observed monthly averaged precipitation time series. CSD indicators are calculated for rolling windows of width *w* = 20 years. The trend is determined by Kendall τ of the respective indicator of the detrended and deseasoned precipitation time series at every site. Stippling marks regions with significantly increasing trends (*P* < 0.05; see Materials and Methods for details on the statistical test and fig. S6). Large parts of southwestern Amazonia show a simultaneous increase in variance and autocorrelation. Mean wet season wind fields (1979 to 2019) at 750 hPa are delineated in grey. The nonlinear trend of the underlying precipitation time series is removed via STL (*95*) with a trend smoother length of 5 years and seasonal smoother length of 13 months. The data are taken from the ERA5 reanalysis (*59*). In particular, the regions with high lag-one autocorrelation correspond well with the main atmospheric moisture transport routes in South America and particularly the aerial river across the Amazon to the subtropics. (**C**) Variance of the spatially averaged precipitation time series (black, left *y* axis) and variance of the spatially averaged, detrended, and deseasoned precipitation time series (blue, right *y* axis) for southwestern Amazonia from 1979 to 2019, calculated in a window *w* = 25 years [4°S to 12.5°S, 62.5°W to 72.5°W, white box in (A) and (B)]. Dashed lines denote linear regressions of the respective time series. For the detrending method, see Materials and Methods. (**D**) Same as (C) for autocorrelation at lag one [AC(1)]. See figs. S7 and S8 for corresponding results using alternative gauge- and satellite-based rainfall datasets.

As for our simulations, we also analyze the precipitation rates from 1979 to 2019 in the ERA5 data. We find an increase of both statistical indicators, variance and lag-one autocorrelation, in large parts of tropical South America (Fig. 6). The variance increases in most parts of Amazonia south of the Amazon River, while the lag-one autocorrelation shows the most pronounced increase in southwestern Amazonia and further to the south. We identify southwestern Amazonia as a region that shows a clear, simultaneous increase in both variance and lag-one autocorrelation, with *P* < 0.1 (figs. S5 and S6). Consequently, the variance and autocorrelation of the averaged precipitation time series in southwestern Amazonia show a clear increase (Fig. 6, C and D). The changes in lag-one autocorrelation and variance inferred here from the ERA5 reanalysis are consistent with corresponding results inferred from independent satellite- and gauge-based rainfall products (figs. S7, B to D, and S8, B to D). In particular, the trends in the autocorrelation show very similar spatial distributions across datasets. We note that increasing variance and particularly increasing lag-one autocorrelation can be observed along the aerial river that provides the main atmospheric moisture transport from the Amazon southward to the South American subtropics (*69*, *70*), suggesting that the continental-scale circulation system is losing stability. According to our results, this loss of stability can be at least partly attributed to the impacts of changes in the Amazon vegetation system, including deforestation, drought-induced degradation, and forest fires.

## DISCUSSION

The revealed changes in the ERA5 data show a substantial similarity to our model simulations for times before crossing the critical deforestation threshold. Alongside the statistical precursor signals, the observed decadal-scale decrease in soil moisture (Fig. 5A) together with the observed dry season prolongation (Fig. 5B), and especially the later wet season onset are consistent with the corresponding transition precursor signals found in our simulations and thus suggest that the coupled atmosphere-vegetation system of the Amazon approaches the theoretically suggested tipping point. The increases in DSL and the later wet season onset in the ERA5 data are in good agreement with the results of previous studies (*25*, *29*, *64*–*67*).

While, in the ERA5 data, the extension of the dry season is mostly observable in the southeastern regions, where deforestation rates are strongest, a recent study (*58*) reports an intensification of the dry season mainly in central and southwestern Amazonia. However, it is concluded there that global climate change and local deforestation effects are the main contributors, while, based on our model results, we additionally suspect circulation changes in response to deforestation in eastern Amazonia as cause for the observed increases of the DSL. In accordance with our results, trends in the atmospheric circulation in tropical South America over the past decades show a higher occurrence of wintertime circulation patterns and decrease of the frequency of circulation patterns characteristics for the transition season, as well as a weakening of the trade winds during and preceding droughts (*27*, *44*). These changes are characterized by increased southerly wind anomalies from the Amazon Basin toward Southeastern South America and intensified southerly cross-equatorial winds; both have been associated with a delayed onset of the monsoon (*44*). These changes in circulation patterns during the transition season have recently been associated with deforestation linked to the absence of deep convection over deforested regions before the wet season onset (*42*). Synoptic scale circulation changes, driven by continued extensive deforestation, could even lead to a delay in the onset of the wet season by more than a month (*71*). Wet season onset estimations based on outgoing longwave radiation support our findings of increasing DSL mainly in southeastern Amazonia and some parts of southwestern Amazonia (*25*, *44*).

Previous modeling studies showed that extensive deforestation of the Amazon rainforest leads to changes in the energy balance of the whole Amazon basin and in the atmospheric circulation over tropical South America, which, in turn, leads to weakened moisture flux and precipitation in these regions (*72*). Deforestation leads to a local reduction of precipitation and evapotranspiration, as well as a decrease in average latent heat release that, in turn, weakens moisture convergence over western Amazonia (*72*). This leads, most likely, to a prolongation of the dry season in these parts of South America.

In accordance with our model results, we propose the following chain of mechanisms in the coupled atmosphere-vegetation dynamics on the way to the deforestation-induced transition. The time needed to initiate the wet season prolongs as the atmospheric moisture and, hence, the average latent heat over the Amazon decreases with proceeding forest loss in the east. Large parts of Amazonia show a negative trend in atmospheric moisture content over the past decades (*25*). The heating gradient between the Atlantic Ocean and the continent weakens and impedes the annual transition into the wet season (*44*). Consistently, we observe increasing DSL and later wet season onset in the ERA5 data (Fig. 5B). The shortened wet season and the decreasing rainfall rates result in lower soil moisture levels. Evapotranspiration rates, however, stay at a high level and further deplete the soil during the dry season. This leads to an all-year-round decrease of the soil moisture and an increasing SMD before the dieback of the rainforest. This increasing SMD can be clearly observed in the ERA5 data (Fig. 5A). After the dieback of the rainforest, the soil moisture approaches an alternative equilibrium consistent with the reduced precipitation and evapotranspiration of the savanna vegetation. At the critical point, the atmospheric moisture content does not suffice anymore to maintain precipitation and, thus, latent heating rates that could switch the system back into the annual wet season, resulting in a permanent dry season state. Consequently, this would result in a dieback of substantial parts of the rainforest.

We emphasize that the decreasing soil moisture that we observe in the ERA5 data is not simply a linear response to decreasing rainfall rates in Amazonia but rather a consequence of the changing hydrological budget of the coupled atmosphere-vegetation system. In general, the soil moisture shows a delayed response to reduced precipitable water and has been shown to be correlated to tropical Atlantic sea surface temperatures and reduced moisture inflow from the Atlantic and northern Amazonia (*27*). The precipitation rates in large parts of Amazonia do not consistently decrease with the negative trend in the soil moisture (fig. S9), and the links between soil moisture, precipitation, and other factors remain uncertain, especially in deforested areas (*27*).

It should also be noted that it is inherently hard to measure the soil moisture in the Amazon rainforest due to the high vegetation coverage that hinders satellite measurements. However, previous studies showed an agreement between satellite-based soil measurements and the ERA5 reanalysis in large parts of Brazil (*27*, *73*). A recent soil moisture reconstruction study that combines satellite and tree ring measurements also supports the strong decline of the soil moisture in the Andes region in the past decades that is visible in the ERA5 data (*74*), increasing the general confidence in the ERA5 soil moisture product.

Similarly, quantifying rainfall in the Amazon region presents difficulties due to the scarcity of rain gauges (*75*). Rainfall products can differ substantially in South America on both spatial and temporal scales (*76*, *77*). While the spatially averaged ERA5 precipitation rates in South America are in good agreement with the gauge-based rainfall product provided by the Global Precipitation Climatology Centre (GPCC) (*78*) after the year 1980, the ERA5 precipitation in tropical South America shows some deviation from GPCC (*79*, *80*). To account for this uncertainty in the ERA5 precipitation, we repeat the statistical analysis for the three different satellite- and gauge-based rainfall products: Climate Hazards Group InfraRed Precipitation with Station data (CHIRPS) (*81*) and GPCC and Global Precipitation Climatology Project (GPCP) (*82*). While the four datasets not necessarily agree on the trend of rainfall over the past four decades (fig. S9), we find a strong agreement in the trends of the statistical CSD indicators (figs. S7 and S8).

The impacts of a weakened atmospheric circulation are expected to be greatest in the southwestern parts of the rainforest, due to the cascading effect of reduced moisture recycling over the basin. This implies that increasing deforestation rates in eastern Amazonia could lead to an extensive reduction of rainfall in remote parts of the rainforest (*72*). Large parts of the rainforest in southwestern Amazonia are still intact, which suggests that our findings of changes in the large-scale precipitation patterns cannot be ascribed to local deforestation effects.

While the characteristic changes in our model simulations can by design be exclusively attributed to forest loss, this is not possible in observational or reanalysis data. Besides forest loss, several competing processes influence the climate in South America, such as El Niño events, sea surface temperature anomalies in the tropical Atlantic, and the consequences of global warming. For example, a poleward shift of the subtropical jets and equatorial contraction of the ITCZ is expected in a warmer climate, possibly leading to a delayed wet season onset and savanna expansion in the southern Amazon (*83*, *84*). It has been shown that deforestation can enhance surface wind acceleration and increase the moisture inflow from the Atlantic and increase precipitation in intact regions of the rainforest due to decreased roughness length (*85*). While we do not account for this effect in our model, it could substantially increase the precipitation throughout the intact regions of the rainforest with further deforestation. Including this effect would potentially reduce the amplitude and abruptness of the transition shown in Fig. 2. Furthermore, an intensification of the Hadley and Walker cells, associated with anomalous sea surface temperatures in the tropical Pacific and north tropical Atlantic, as well as reduced moisture transport from the tropical Atlantic Ocean, has partially been linked to an increase of the frequency of dry days during the transition season in southern Amazonia over the past decades (*24*–*26*, *86*).

Our model simulations imply that all four indicators will occur consistently before the critical transition, and, correspondingly, we reveal that all four of them occur consistently in the ERA5 data and in other observation datasets. It is unlikely that low-frequency natural variability such as the atlantic multidecadal oscillation (AMO) and El Niño–Southern Oscillation (ENSO), possibly in conjunction with anthropogenic climate change, would lead to a similarly consistent occurrence of all four—in principle, independent—indicators. Moreover, we test the trends in the CSD indicators based on a phase surrogate test that preserves the variance and the autocorrelation structure of the original time series (fig. S6). In this sense, the detected trends are statistically significantly positive given the “natural variability” (in terms of variance and autocorrelation) and can thus not be explained by natural variability.

We showed that forest loss, caused by direct deforestation, droughts, and fires, might vastly contribute to a changing climate in Amazonia and could drive the coupled rainforest-monsoon circulation system past a tipping point. Recent changes in precipitation patterns, increasing DSL, reduced soil moisture, and more frequent extreme events might be much stronger linked to deforestation than previously assumed. The results presented here suggest an upcoming regime shift of the Amazon ecosystem if deforestation is not brought to a halt. We outline a detailed analysis of the observed changes in the hydrological variables that we identify with physical and statistical precursor signals, using high-resolution coupled vegetation-atmosphere models, as an urgent subject of future research.

## MATERIALS AND METHODS

### Model design

The main focus of the model is the relationship between a deforestation-induced decrease in the total surface heat flux and a positive feedback mechanism associated with the release of latent heat. To investigate the consequences of deforestation, a nonlinear moisture transport model along a one-dimensional trajectory from the mouth of the Amazon River to the western boundary of the Amazon basin, following the low-level winds in the monsoon season, is constructed (see Fig. 1). The underlying equations are given by the moisture balance equations

$$\partial_{t}A=E-P-\nabla \cdot M$$ (1)

$$\partial_{t}S=P-E-R$$ (2)

where *A* denotes the atmospheric moisture content, *E* is evapotranspiration, *P* is precipitation, *S* is the total soil moisture content, and *R* is the runoff. The term ∇ · **M** denotes the divergence of vertically integrated moisture flow and is defined at each atmospheric layer λ as ∇ · **M**^λ^ ≔ *A*^λ^**W**^λ^, where **W**^λ^ is the respective wind speed. The runoff *R* and the precipitation *P* are modeled as effective functions of *S* and *A*, respectively, with functional dependencies estimated from the ERA5 reanalysis. The evapotranspiration is obtained by sampling from a three-dimensional estimated probability density, dependent on the soil moisture *S* and the surface net solar radiation. The winds **W** = **W**^T^ + **W**^H^ are modeled in terms of a trade wind component **W**^T^ and a convective latent-heating component **W**^H^. **W**^H^ represents the amplification of wind speeds due to condensational heating in the wet season. Because this heating is dependent on precipitation and, hence, atmospheric moisture itself, the heating component introduces the nonlinearity to the model. The wind is modeled as

$$W_{i}(t)=W_{i}^{T}(t)+W_{i}^{H}(t)$$ (3)

$$W_{i}^{T}(t)=(w_{0}-w_{c})\left(1+\frac{1}{1+e^{w_{1}(t)\cdot i-w_{2}}}\right)+W_{i>70}^{\mathrm{dry}}(t)$$ (4)

$$W_{i}^{H}(t)=w_{c}L\pi (t)\left(1+\frac{1}{1+e^{w_{1}(t)\cdot i-w_{2}}}\right)$$ (5)

Here, π(*t*) denotes the heating gradient between the Atlantic Ocean and the trajectory

$$\pi (t)={\left\langle H\right\rangle }^{\mathrm{trajectory}}(t)-{\left\langle H\right\rangle }^{\mathrm{AO}}(t)$$ (6)

with *H* = *H*^sensible^ + *H*^latent^ + *H*^rad^ and ⟨·⟩*^R^* denoting the spatial average over the region *R*. The coefficients *w*_0_, *w*_1_(*t*), and *w*_2_ are adjusted such that the modeled winds in the wet season match the observed wind speeds. Here, the parameters are chosen to be

$$w_{0}=16.5\;\mathrm{km}/\mathrm{hour}$$ (7)

$$w_{1}=0.6-[0.03\cdot \cos\left(\frac{2\pi }{8760}t-\pi \right)-0.03]$$ (8)

$$w_{2}=3.4$$ (9)

The parameter *w_c_* and *w*_0_ determine the strengthening of the wind speeds and moisture inflow due to the latent heat release over the Amazon during the wet season. The amplification factor (AF) of the winds is then given by

$$\mathrm{AF}=\frac{w_{0}}{w_{0}-w_{c}}$$ (10)

Varying *w_c_* thus corresponds to controlling the strength of the heating-related feedback. The dimensionality factor *L* is defined as

$$L=\max\left\{\frac{1}{\pi (t)}\right\}$$ (11)

and calculated for the undisturbed case, with no deforestation. In other words, *L* ensures that the amplification due to the heating winds is limited by AF. Only wind speeds at the 750-hPA level are considered because they are very similar to the mean wind speeds on the 700- to 900-hPa levels.

We integrate the discretized equations

$$A_{i}(t+1)=A_{i}(t)+E_{i}(t)-P_{i}(t)-\frac{W_{i}(t)A_{i}(t)-W_{i-1}(t)A_{i-1}(t)}{l}$$ (12)

$$S_{i}(t+1)=S_{i}(t)+P_{i}(t)-E_{i}(t)-R_{i}(t)$$ (13)

along the 100 boxes of the trajectory, in hourly steps. The subscript *i* denotes the respective spatial box, and *l* = 30 km is the length of a single box. The moisture divergence ∇ · **M** is realized as forward difference quotient. Initial conditions are derived from the ERA5 reanalysis (*59*). We validate the model results against the ERA5 data (see fig. S1). For the investigation of precursor signals, we add white noise (σ = 0.1) to each *A_i_*(*t*) in Eq. 12.

Deforestation is simulated as a 40% reduction of the evapotranspiration *E* and a 40% increase in the sensible heat. However, the latent heat flux is two to four times higher than the sensible heat flux. This means that the reduction of *E* is the determining change. In addition to this ad hoc deforestation, a feedback mechanism between higher plant water stress and the vegetation is implemented. Less precipitation and longer dry seasons due to the weakening of the latent heat feedback lead to less soil moisture, which will eventually lead to increased tree mortality. Here, a simple threshold model is proposed. An integrated SMD is dynamically calculated, and, when the SMD exceeds a set threshold, this leads to deforestation of the box. For every box, the lowest soil moisture value before deforestation is set as a baseline. The integrated SMD is the sum of the soil values below the baseline. If the deficit exceeds the threshold, then the same consequences as in the case of manual deforestation occur.

### Dry season length

There exist several methods to determine the onset and demise of the wet season in tropical regions. Here, we use three different methods to determine the DSL in the ERA5 data.

First, we calculate the climatological mean rainfall rate $\bar{P}$ and the running 30-day mean precipitation rate ${\bar{P}}_{30d}$ for all years. We identify all days where ${\bar{P}}_{30d}$ is below the climatological mean and determine the longest sequence of consecutive dry days, ignoring wet spells (days with ${\bar{P}}_{30d}]>\bar{P}$) shorter than 10 days. The longest sequence of dry days is the DSL of the respective year. The wet season retreat is the first day of the consecutive dry days and the onset date is the last day.

The second method is based on Liebmann *et al.* (*87*, *88*). We calculate the 5-day mean precipitation for all years and determine the annual cumulative pentad rainfall anomaly *A*(*d*) for every pentad *d*

$$A(d)=\sum_{j=0}^{d}(P_{j}-\bar{P})$$

with *P_j_* as rainfall on pentad *j* and $\bar{P}$ as climatological mean rainfall rate. The anomaly *A*(*d*) increases when the pentad rainfall is above the climatological mean and decreases otherwise. The onset date of the wet season is then the maximum of *A*(*d*), and the minimum of *A*(*d*) denotes the end of the wet season. The difference between demise and onset yields the DSL.

The third method is a modification of Marengo *et al.* (*29*, *89*). We calculate the 5-day mean precipitation for every year, ${\bar{P}}_{5d}$. The wet season demise is determined by the first date when ${\bar{P}}_{5d}$ changes from above to below the climatological annual mean rain rate during six of the eight pentads. Vice versa, the wet season onset is the first pentad when ${\bar{P}}_{5d}$ changes from below to above the climatological annual mean rain rate. If this criterion fails for a specific year, then the condition is relaxed to five of the eight pentads and, ultimately, to four of the eight pentads.

### CSD and associated precursor signals

Several large-scale components of the Earth system are suspected to show abrupt shifts between different states when important control parameters, such as temperature or CO_2_, approach critical thresholds, so-called tipping points (*1*). Key examples of such potential tipping elements are given by the Atlantic Meridional Overturning Circulation, the polar ice sheets, and the Amazon rainforest. While it is, in principle, hard to predict these abrupt shifts, various precursor signals that precede bifurcation-induced transitions in low-order random dynamical systems have been proposed (*36*, *90*, *91*). When the system approaches the critical threshold, the state of the system resembles more and more its past states, which is known as CSD. The restoring forces of the system diminish, and the rate of return to equilibrium after a perturbation approaches zero. This loss of resilience can be seen as a rise in the lag-one autocorrelation and variance of the fluctuations before crossing the tipping point (*36*, *92*). Under the assumption that leading dynamical models of Earth system tipping elements can, to a first approximation, be represented by low-order random dynamical systems, the above motivates to search for precursor signals in the dynamics of tipping elements. Precursor signals related to CSD have been identified, for example, before collapses of the Atlantic Meridional Overturning Circulation in comprehensive model simulations (*93*, *94*).

We calculate the variance and lag-one autocorrelation of the detrended time series within endpoint rolling windows half the size of the reanalysis time series (*w* = 20 years). For our model time series, we calculate the CSD indicators in a rolling window of size *w* = 150 years from year 0 to year 200 (Fig. 4).

We detrend the time series via seasonal trend decomposition (STL) based on locally estimated scatterplot smoothing (LOESS) (*95*) with the statsmodels Python package (*96*). The particular detrending parameters are given in the figure captions. The lag-one autocorrelation is computed directly as Pearson correlation coefficient between the time series and itself shifted by one time step. It should be noted that both variance and lag-one autocorrelation have to increase (significantly) to conclude the proximity to a critical transition (*90*, *97*).

### Significance testing

To test the significance of our results, we test against the hypothesis that the trends in the CSD indicator time series are due to chance. By randomly shifting the phases in Fourier space, we generate *n* = 10,000 surrogates of the time series of the same length, which have no trend and preserve the variance and spectrum, and, hence, the autocorrelation function of the original time series (*98*–*100*). We calculate Kendall’s τ for each generated surrogate time series and compare it with the Kendall τ of the original data. The fraction of cases where τ of the surrogates was equal or greater than the original τ gives the *P* value of our significance test, for a given τ value.

### Sensitivity analysis

The following simulation parameters are varied to investigate the sensitivity of our results to these choices: the AF, the heating over the tropical Atlantic Ocean ⟨*H*⟩^AO^(*t*), the reduction of the evapotranspiration after deforestation Δ*E*, and the integrated SMD threshold. We find that our results are robust against reasonable variations in these parameters.

The consequences of deforestation depend on the values chosen for AF and ⟨*H*⟩^AO^(*t*). The AF determines the strength of the heating-related feedback; the higher the AF, the more severe the consequences. Nevertheless, for all AF within the bounds set by previous studies (*46*–*48*), we find an abrupt dieback of the rainforest (fig. S10).

A higher average heating over the Atlantic Ocean leads to a lower heating gradient between ocean and continent and, therefore, to an earlier breakdown of the heating-related feedback. We vary the spatial box used for averaging the atmospheric heating over the Atlantic by 1° to 2° in every direction and find 〈*H*〉^AO^ = 80 W/m^2^ to 〈*H*〉^AO^ = 140 W/m^2^ during the wet season (December to February) and 〈*H*〉^AO^ = 30 W/m^2^ to 〈*H*〉^AO^ = 80 W/m^2^ during the dry season (June to August), with an annual amplitude of 20 W/m^2^ to 50 W/m^2^. We use 〈*H*〉^AO^ = 80 W/m^2^, 〈*H*〉^AO^ = 100 W/m^2^, 〈*H*〉^AO^ = 120 W/m^2^, and 〈*H*〉^AO^ = 140 W/m^2^ for our simulations, each with an annual amplitude of 20 and 40 W/m^2^. We show the precipitation rates for different AF and *H*^AO^ after successive deforestation in fig. S10. Although the position of the critical deforestation threshold and the abruptness of the transition depend on these choices, the results remain qualitatively unaltered.

The integrated SMD threshold is an artificial parameter that is chosen such that prolonged drought conditions over multiple years lead to degradation of the rainforest, which we quantitatively implement in the same way as deforestation. The specific choice for the SMD determines the timing of the rainforest dieback, but it has no influence on the severity of the rainfall decline (see fig. S11A). To investigate the statistical precursor signals and trends in the CSD indicators, we choose the SMD such that the dieback of the rainforest happens after 50% deforestation rate.

The reduction of the evapotranspiration Δ*E* is an essential parameter that determines the strength at which deforestation and vegetation degradation due to a persistent SMD affect the coupled hydrological cycle. The specific choice of the reduction, hence, influences the timing of the dieback. The higher Δ*E*, the lower the precipitation rates after deforestation and the earlier the dieback. Results from a field study suggest reductions of around 40% for deforestation/degradation from closed rainforest to savanna, crop, or pasture (*60*). We show the influence of Δ*E* on the wet season precipitation for Δ*E* = 30 to 50% for exemplary AF and ⟨*H*⟩^AO^(*t*) in fig. S11B. Qualitatively, the results are similar, however, with a rainforest dieback eventually occurring for all Δ*E*.
