# Supplementary material for: The South American monsoon approaches a critical transition in response to deforestation
Source: Sci Adv. 2023 Oct 4;9(40):eadd9973. doi: 10.1126/sciadv.add9973 (PMC10550231; doi:10.1126/sciadv.add9973)
Supplement: Supplementary file 1 — Figs. S1 to S11 [file sciadv.add9973_sm.pdf]

Science Advances
AAAS

Supplementary Materials for

The South American monsoon approaches a critical transition in response to deforestation

Nils Bochow and Niklas Boers

Corresponding author: Nils Bochow, nils.bochow@uit.no

Sci. Adv. **9**, eadd9973 (2023)
DOI: 10.1126/sciadv.add9973

This PDF file includes:

Figs. S1 to S11

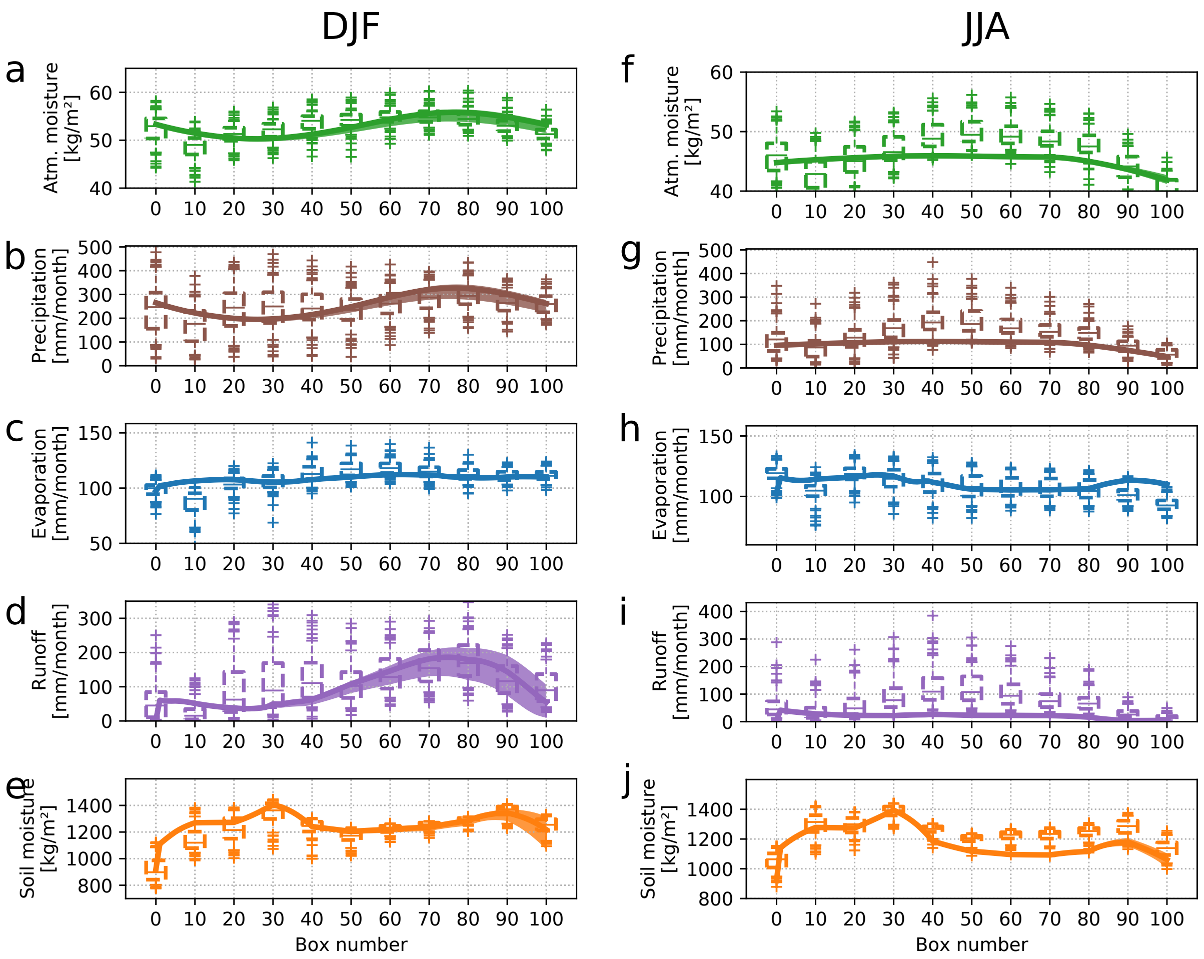

Figure S1: Comparison between model results and reanalysis. Comparison between ERA5 reanalysis (box plots dashed with whiskers at 5th an 95th percentile) and model results (solid lines, filled area) for all atmospheric variables of the model, along the trajectory of 100 boxes. Results are shown for the wet season (DJF, **a-e**) and for the dry season (JJA, **f-j**). Filled areas indicate the range of values for all choices of the different simulation parameters AF and $\langle H\rangle^{\mathrm{AO}}$. Solid line denotes mean value. Note that we use all available soil moisture layers in ERA5 with a total depth of 2.89 m.

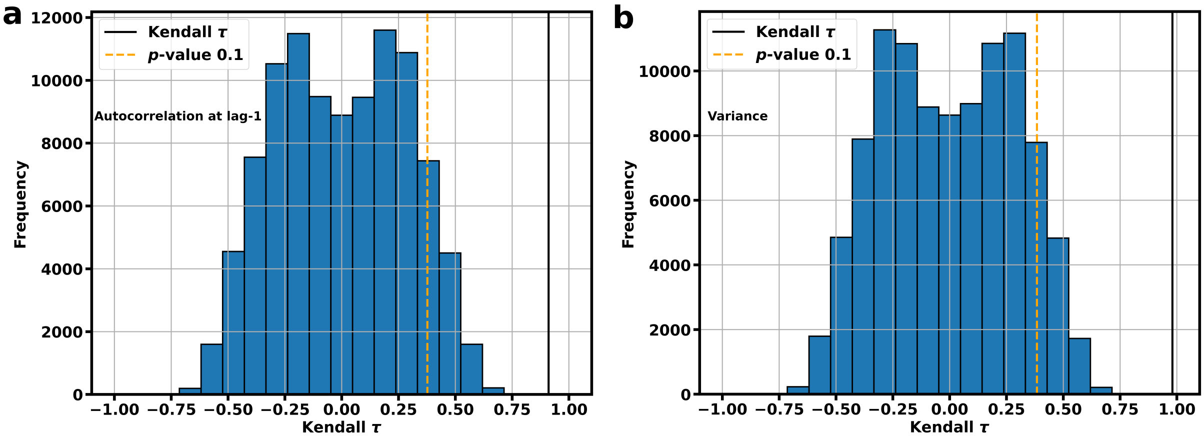

Figure S2: Histogram of Kendall τ derived from 100,000 surrogate time series of lag-one autocorrelation and variance of the modelled precipitation rates. **(a)** Histogram of lag-one autocorrelation from surrogate time series generated by phase randomisation (see Methods). The dashed orange line denotes the threshold corresponding to a p-value of $p = 0.1$, and the black line denotes Kendall τ derived from the original modelled time series series (see Fig. 4 a). **(b)** Same as **(a)** but for variance.

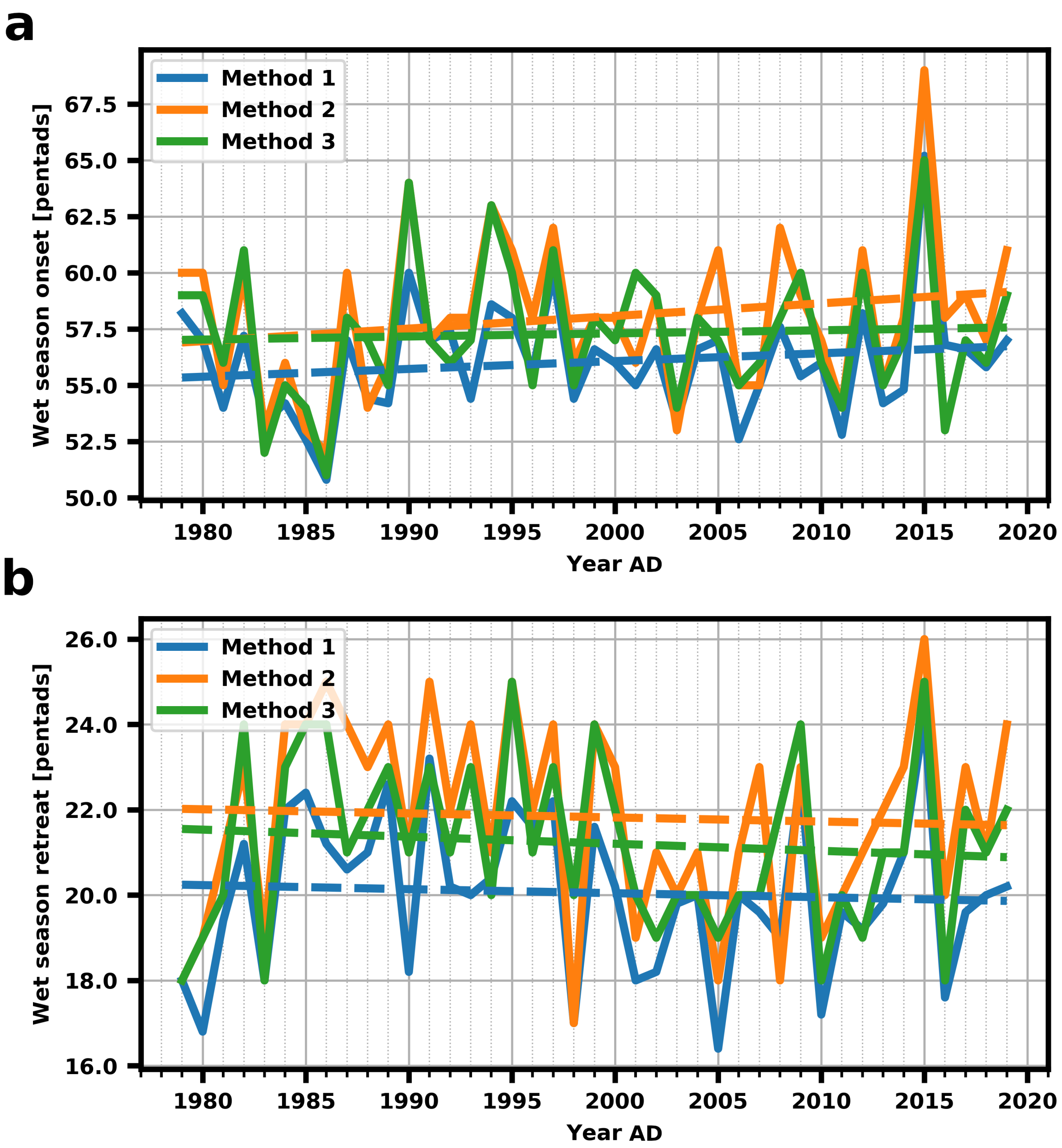

Figure S3: Wet season onset and retreat date in southern Amazonia. (a) Wet season onset date in southern Amazonia ($5-15°$S, $50°-70°$W) from 1979-2019 for the three different methods used (see methods). Dashed lines denote the corresponding linear trends. We find an increase of 3.5 (blue), 5.6 (orange) and $1.4\,pentad$/century (green) for the three methods, respectively. **(b)** Same as **(a)** but for the wet season retreat date. We find a decrease of -0.9 (blue), -1.0 (orange), and $-1.6\,$pentad/century (green) of the retreat date. Data is taken from the ERA5 reanalysis (*58*). The wet season hence tends to initiate later and end earlier, consistently with an increase in DSL.

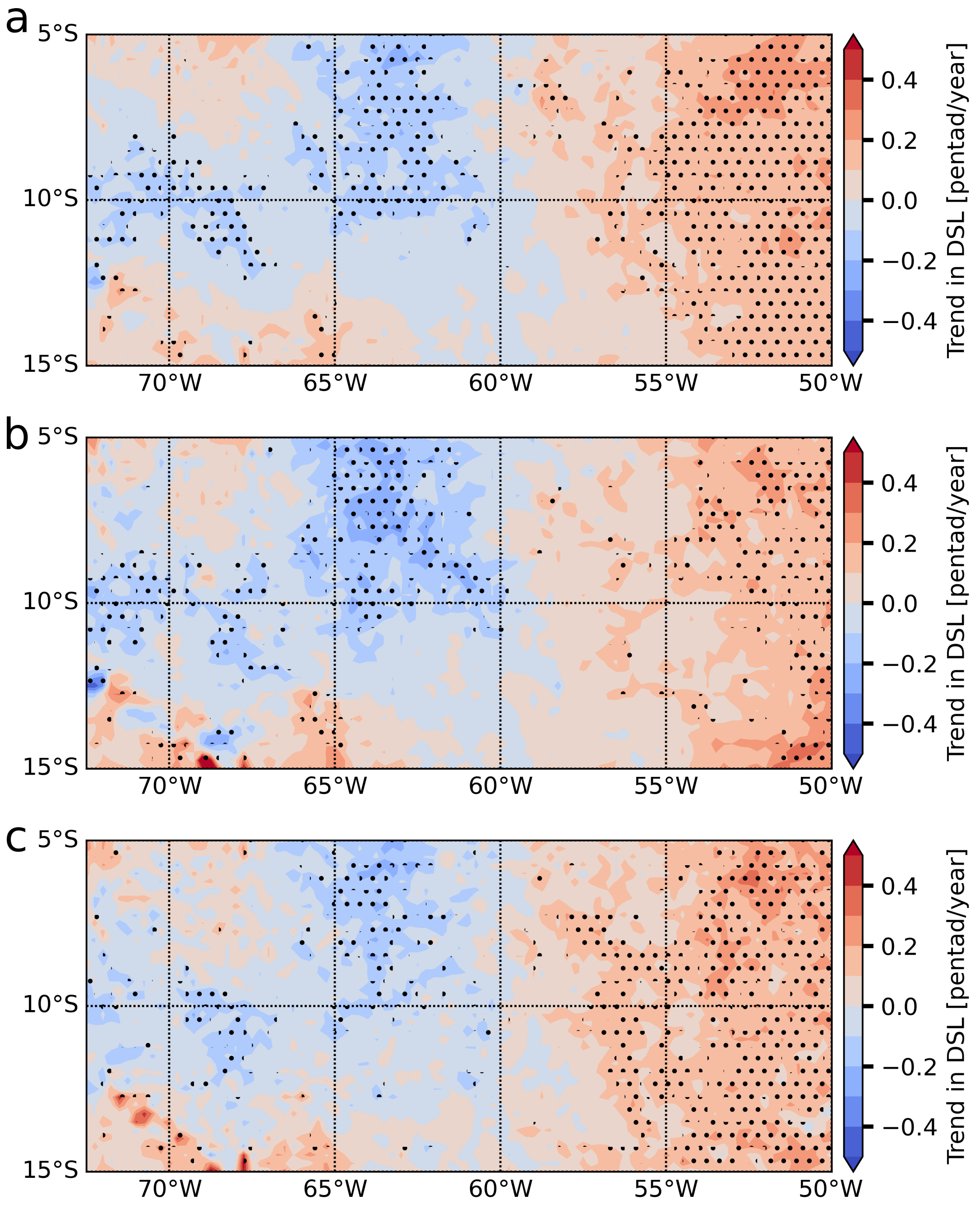

Figure S4: Map of trends in dry season length for the southern Amazon in ERA5. Calculated linear trend in the dry season length from 1979-2019 in southern Amazonia, based on the ERA5 reanalysis, using the three different methods used (see methods). Areas with p-value < 0.05 are hatched. **(a)** Dry season length calculated with method 1, **(b)** Calculated with method 2, **(c)** calculated with Method 3. All three methods show the same spatial pattern.

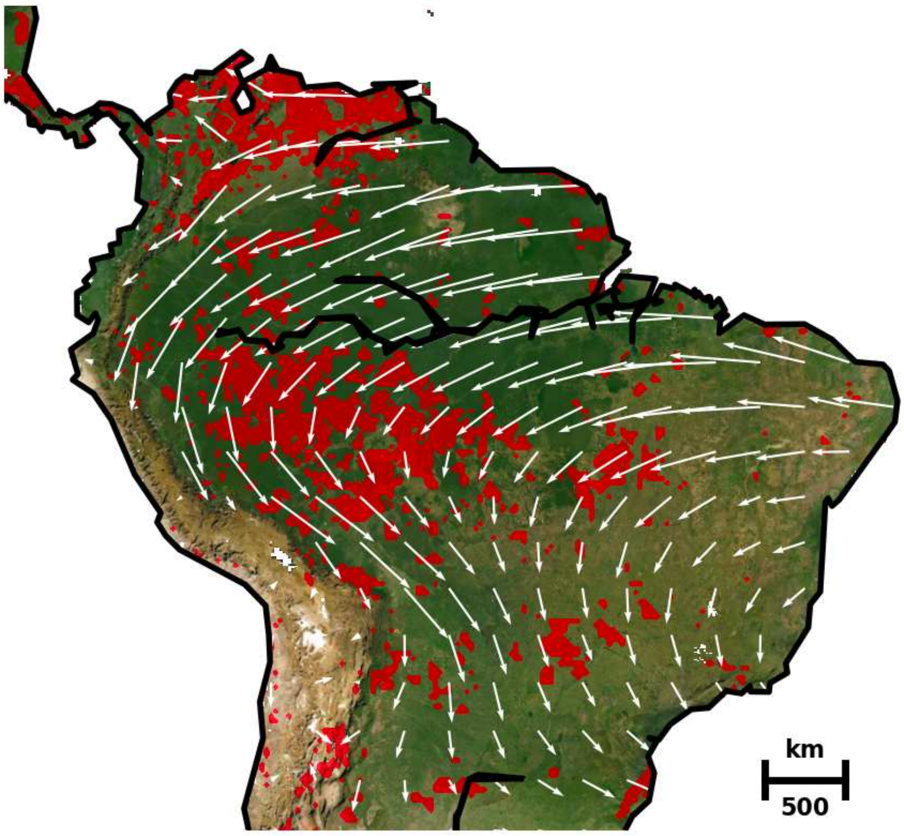

Figure S5: Regions with simultaneous increase in variance and autocorrelation in ERA5. The red areas denote regions where variance and autocorrelation in ERA5 precipitation rates increase. See Fig. 6 and method section for methods.

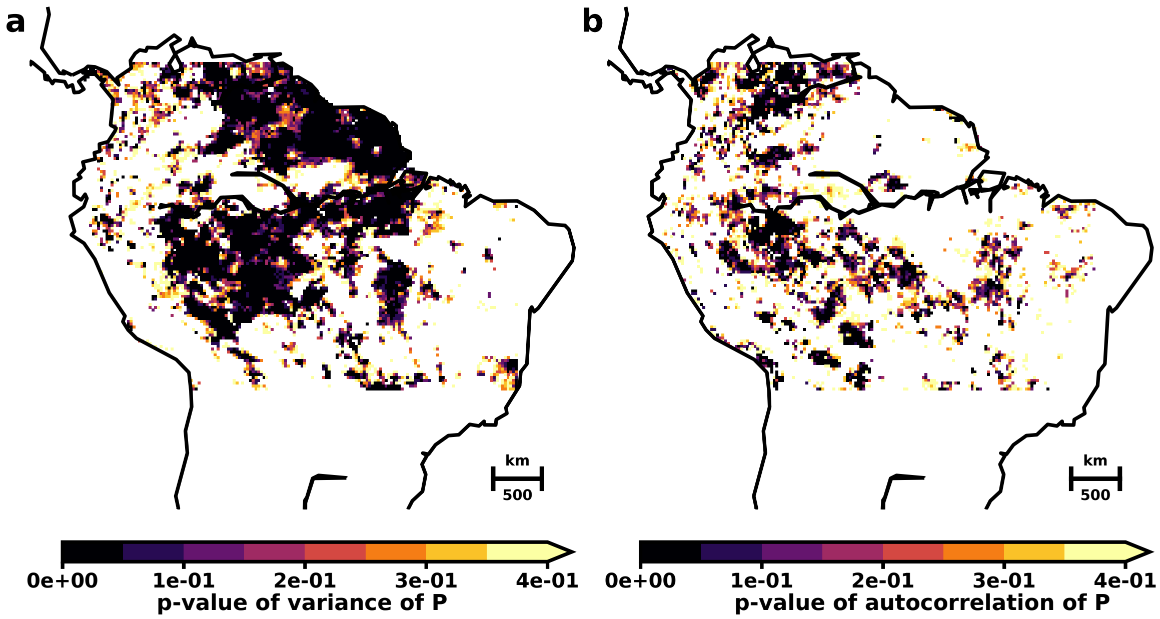

Figure S6: Map of p-values estimated from distribution of Kendall τ derived from 10000 surrogate data sets of autocorrelation and variance in southwestern Amazonia in ERA5. **(a)** Map of the p- values for our phase-surrogate test to determine the significance of the trend in the lag-one autocorrelation in the precipitation rates in northern South America. The p-values are only shown for regions with positive trend in the autocorrelation. White areas denote regions with negative trends. **(b)** Same as **(a)** but for the variance. It can be inferred that the increases in both variance and lag-one autocorrelation in southwestern Amazonia are mostly significant.

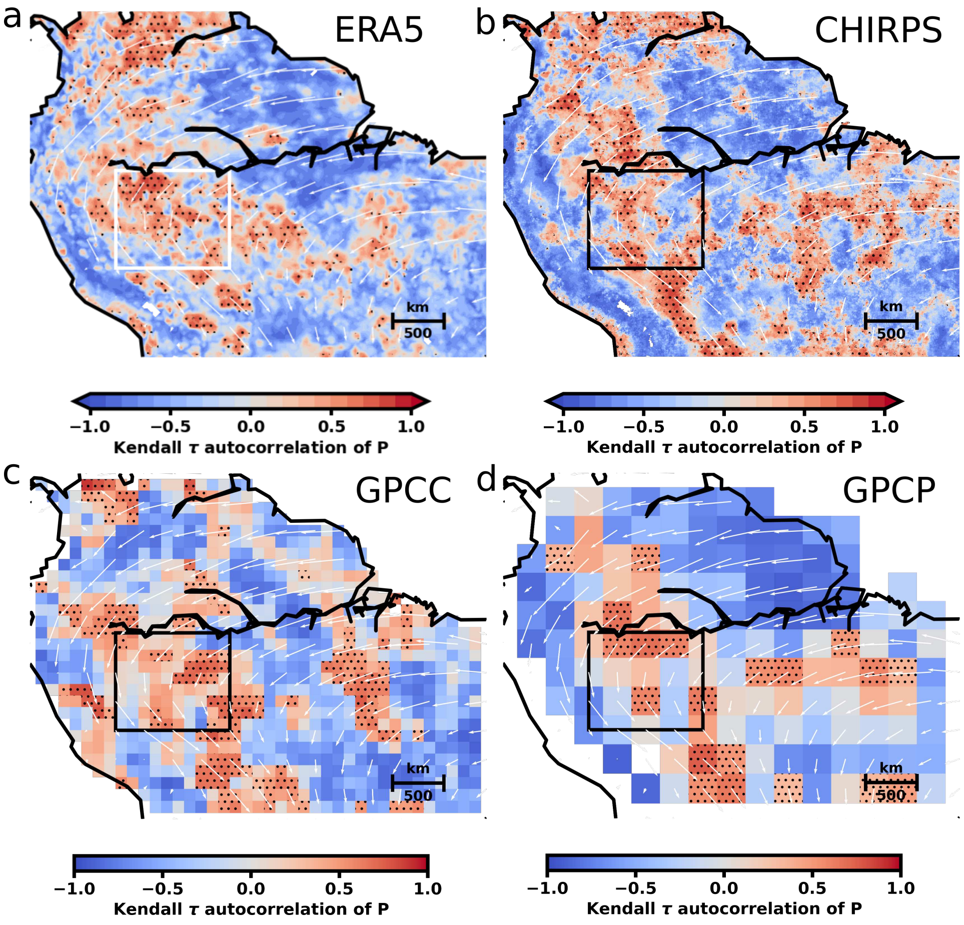

Figure S7: Temporal lag-one autocorrelation in different precipitation data sets. Spatial patterns of Kendall τ for the lag-one autocorrelation of observed monthly averaged precipitation time series for different precipitation data sets. The autocorrelation is calculated for rolling windows of width $w = 20$ years. The trend is determined by Kendall τ of the respective indicator of the detrended and deseasoned precipitation time series at every site. Stippling marks regions with significantly increasing trends ($p < 0.05$, see Methods for details on the statistical test. Mean wet season wind fields (1979-2019) at 750 hPa are delineated in white. The non-linear trend of the underlying precipitation time series is removed via STL (*85*) with a trend smoother length of 5 years and seasonal smoother length of 13 months. **(a)** For ERA5 precipitation (1979-2019). This subpanel is identical with Fig. 6a. **(b)** CHIRPS (1981-2022) (*79*) **(c)** GPCC (1982-2020) (*76*) **(d)** GPCP (1979-2022) (*80*).

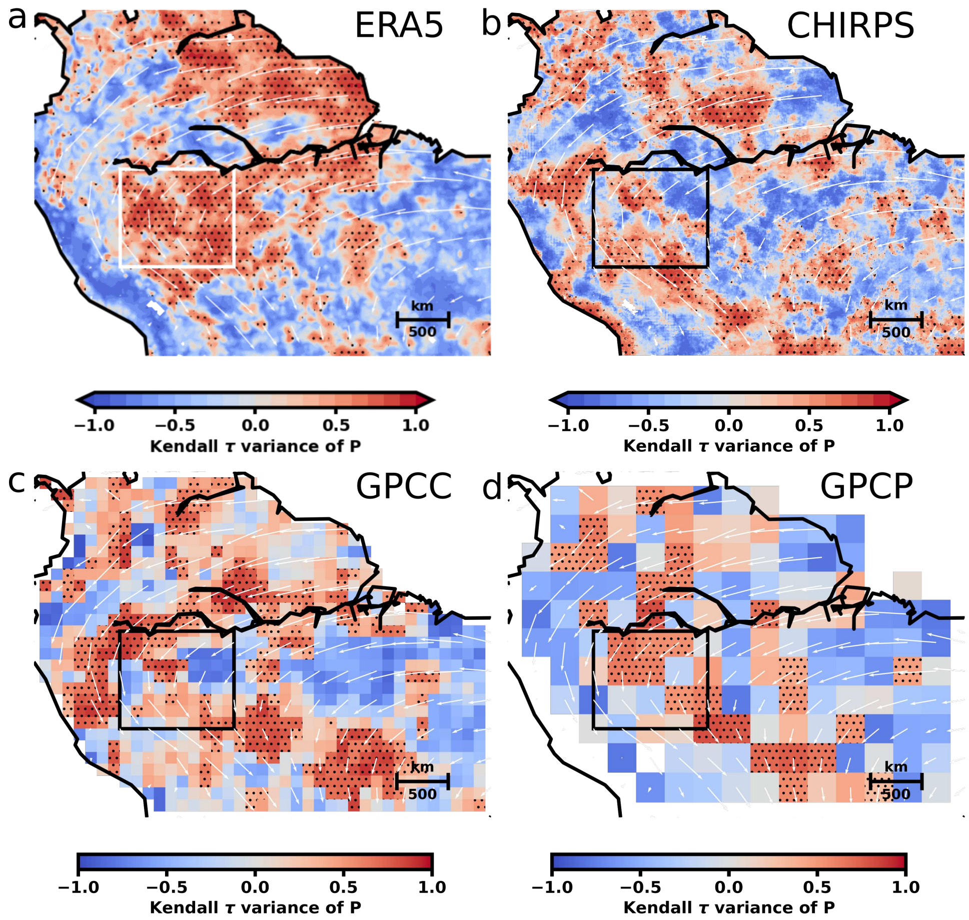

Figure S8: Temporal variance in different precipitation data sets. Spatial patterns of Kendall τ for the variance of observed monthly averaged precipitation time series for different precipitation data sets. The variance is calculated for rolling windows of width $w = 20$ years. The trend is determined by Kendall τ of the respective indicator of the detrended and de-seasoned precipitation time series at every site. Stippling marks regions with significantly increasing trends ($p < 0.05$, see Methods for details on the statistical test. Mean wet season wind fields (1979-2019) at 750 hPa are delineated in white. The non-linear trend of the underlying precipitation time series is removed via STL (*85*) with a trend smoother length of 5 years and seasonal smoother length of 13 months. **(a)** For ERA5 precipitation (1979-2019). This subpanel is identical with Fig. 6b. **(b)** CHIRPS (1981-2022) (*79*) **(c)** GPCC (1982-2020) (*76*) **(d)** GPCP (1979-2022) (*80*).

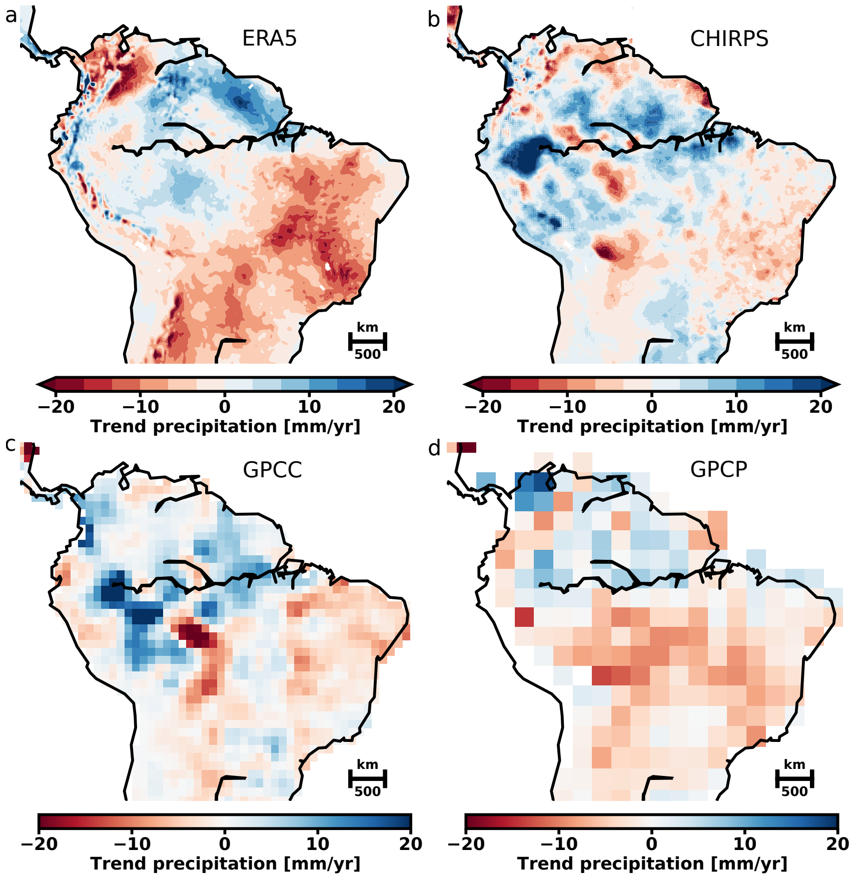

Figure S9: Trends in precipitation rates in South America obtained from different precipitation data sets. **(a)** Calculated linear trend in yearly averaged precipitation rates from 1979-2019 in South America, determined from the ERA5 reanalysis. Large parts of the Amazon rainforest show a positive trend (blue) in the precipitation rates. This suggests that the observed decreasing soil moisture is not a linear response to the precipitation rates but rather a response to changes of the hydrological budget of the coupled atmosphere-vegetation system in ERA5. **(b)** Same as **(a)** but for the CHIRPS dataset from (1981-2022). **(c)** GPCC (1982-2020) **(d)** GPCP (1979-2022).

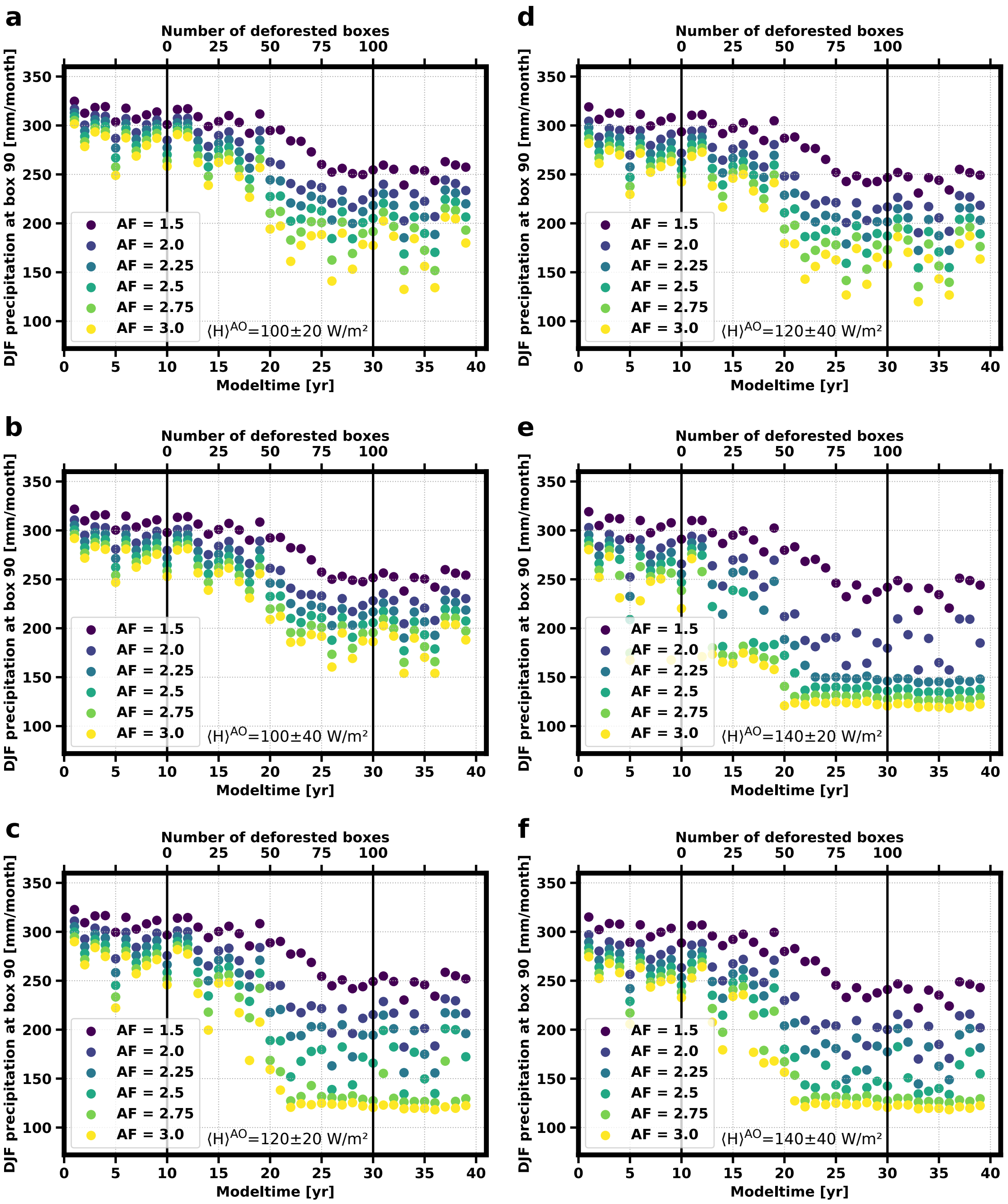

Figure S10: Simulated wet season precipitation for different amplification factors and atmospheric heating over the Atlantic, after successive deforestation. We show the wet season precipitation P_{DJF} in box 90 for all amplification factors AF and a selection of investigated atmospheric heating values over the Atlantic ocean $\langle H \rangle^{\mathrm{AO}}$, with successive deforestation. Deforestation is initiated at year 10 and is completed at year 30. All simulations show a dieback of the rainforest prior to 50% deforestation. For lower values of AF and $\langle H \rangle^{\mathrm{AO}}$ there is still an annual transition into the wet season (**a**, **b**, **d**) after complete rainforest loss. For high AF and $\langle H \rangle^{\mathrm{AO}}$ we find a permanent dry season state after rainforest dieback (**c**,**e**,**f**). It should be noted that the amplification factor is most realistically between 2-3 (*45–47*).

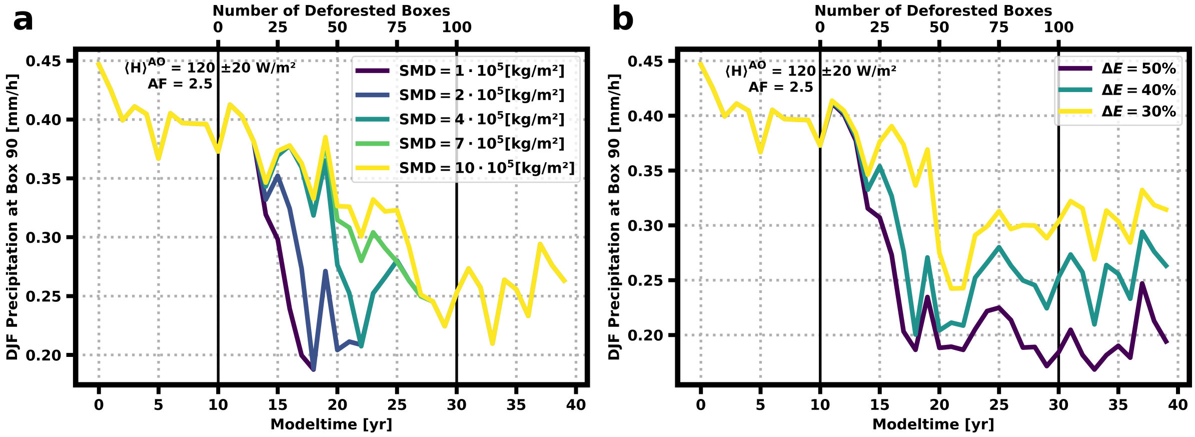

Figure S11: Simulated wet season precipitation for different soil moisture deficit thresholds and reductions of the evapotranspiration after successive deforestation. (**a**) Wet season precipitation P_{DJF} in box 90 for different values of the soil moisture deficit threshold SMD after successive deforestation. The atmospheric heating over the Atlantic ocean $\langle H \rangle^{\mathrm{AO}} = (120 \pm 20)\,\mathrm{W/m^2}$ and the amplification factor $\mathrm{AF} = 2.5$ are fixed. Deforestation is initiated at year 10 and is completed at year 30. All simulations show dieback of the rainforest prior to 50% deforestation. (**b**) Same as (**a**) but for different values of the reduction of the evapotranspiration after deforestation ΔE.
